# Enhancing Processability and Multifunctional Properties of Polylactic Acid–Graphene/Carbon Nanotube Composites with Cellulose Nanocrystals

**DOI:** 10.3390/polym18010099

**Published:** 2025-12-29

**Authors:** Siting Guo, Evgeni Ivanov, Vladimir Georgiev, Paul Stanley, Iza Radecka, Ahmed M. Eissa, Roberta Tolve, Fideline Tchuenbou-Magaia

**Affiliations:** 1 Centre for Engineering Innovation and Research, School of Architecture, Computing and Engineering, Faculty of Science and Engineering, University of Wolverhampton, Wolverhampton WV1 1 LY, UK; s.guo3@wlv.ac.uk; 2 Open Laboratory on Experimental Micro and Nano Mechanics (OLEM), Institute of Mechanics, Bulgarian Academy of Sciences, Acad. G. Bonchev Str. Block 4, 1113 Sofia, Bulgaria; ivanov_evgeni@imbm.bas.bg (E.I.); vgeorgiev@imbm.bas.bg (V.G.); 3 Research and Development of Nanomaterials and Nanotechnologies—NanoTechlab Ltd., Acad. G. Bonchev Str. Block 4, 1113 Sofia, Bulgaria; 4 Center of Competence for Mechatronics and Clean Technologies “Mechatronics, Innovation, Robotics, Automation and Clean Technologies”—MIRACle, 1113 Sofia, Bulgaria; 5 National Centre of Excellence Mechatronics and Clean Technologies, 8 bul. Kliment Ohridski, 1756 Sofia, Bulgaria; 6 School of Architecture, Computing and Engineering, National Brownfield Institute, University of Wolverhampton, Wolverhampton WV1 1 LY, UK; 7 Research Institute of Healthcare Sciences, School of Pharmacy & Life Sciences, Faculty of Science and Engineering, University of Wolverhampton, Wulfruna Street, Wolverhampton WV1 1LY, UK; i.radecka@wlv.ac.uk (I.R.); a.m.eissa@wlv.ac.uk (A.M.E.); 8 Department of Biotechnology, University of Verona, Strada Le Grazie 15, 37134 Verona, Italy

**Keywords:** polylactic acid, carbon nanotubes, graphene nanoplatelets, biobased composite filaments, rheological behaviour, electrical conductivity, thermal conductivity, extrusion, smart packaging, sensors

## Abstract

The growing accumulation of plastic and electronic waste highlights the urgent need for sustainable and biodegradable polymers. However, developing intrinsically conductive biodegradable polymers remains challenging, particularly for packaging and sensing applications. Poly(lactic acid) (PLA) is intrinsically non-conductive, and enhancing its functionality without compromising structural integrity is a key research goal. In this study, PLA-based filaments were developed using melt extrusion, incorporating cellulose nanocrystals (CNCs), graphene nanoplatelets (GNPs), and carbon nanotubes (CNTs), individually and in hybrid combinations with total filler contents between 1 and 5 wt%. The inclusion of CNC enhanced the dispersion of GNP and CNT, promoting the formation of interconnected conductive networks within the PLA matrix, allowing the percolation threshold to be reached at a lower fillers concentration. Hybrid formulations showed a balance melt strength and processability suitable for fused deposition modelling (FDM) 3D printing and prototypes successfully made. This study also provides the first systematic evaluation of temperature-dependent thermal conductivity of PLA-based composites at multiple temperatures (25, 5, and −20 °C), relevant to typical food and medical supply chains conditions.

## 1. Introduction

The demand for sustainable and high-performance materials has intensified due to growing environmental concerns and the global transition toward a sustainable circular economy. Polymers remain indispensable because of their versatility and tuneable properties [1]. However, the extensive use of petroleum-based polymers has raised environmental issues, driving the development of biodegradable, bio-based alternatives to reduce fossil dependence and plastic pollution [2,3]. In this regard, Poly(lactic acid) (PLA) is a biodegradable and renewable thermoplastic derived from natural feedstocks such as corn starch, sugar, or potato [2] with excellent properties including biocompatibility, excellent stiffness, high strength, low coefficient of thermal expansion, and good transparency [4,5]. These have positioned PLA as one of the most widely investigated polymers in different fields, including packaging, biomedical applications, engineering, and additive manufacturing [6,7,8]. PLA is also a leading material for fused deposition modelling (FDM) printing due to its easy processability and compatibility with extrusion-based manufacturing [9]. Moreover, making PLA conductive expands its application to sensors and smart systems such as smart detection devices and packaging [8,10,11]. However, its low impact resistance, heat stability, and drawability, as well as limited barrier properties and inadequate crystallisation kinetics, leading to compromised thermo-mechanical performance hamper its application in demanding or multifunctional environments [4,5,12,13,14,15,16].

It is now recognised that the incorporation of carbon-based nanofillers such as graphene nanoplatelets (GNPs) and carbon nanotubes (CNTs) into a PLA matrix can transform the polymer into a conductive material while simultaneously improving its mechanical and thermal performance. These nanofillers can act as heterogeneous nucleating agents, accelerating PLA crystallisation and promoting the formation of percolated conductive networks. Furthermore, synergistic effects arising from the combination of CNT and graphene have been shown to significantly improve the stiffness, toughness, and multifunctionality of PLA composites, making them suitable for smart and active packaging applications that require mechanical robustness and responsive functionality [17].

Despite these benefits, achieving homogeneous dispersion of carbon nanofillers in PLA remains a major challenge because these fillers tend to agglomerate and show weak interfacial compatibility with the polymer matrix, which compromises their overall effectiveness [18]. Cellulose nanocrystals (CNCs) offer a unique bio-based strategy to address these issues. CNC is renewable, bio-based rod-like nanoparticles with high tensile strength, low density, and high surface area and aspect ratio. It can act as a synergistic filler and morphology modifier [19,20]. CNC incorporation facilitates a more uniform distribution of hydrophobic carbon fillers within PLA through physical mechanisms rather than direct interfacial bonding. Indeed, CNC functions as a nano-spacer that mitigates carbon filler re-agglomeration and promotes the formation of a hybrid network [21,22,23], thereby enhancing interfacial stress transfer and altering the composite’s rheological characteristics [2]. While CNC dispersion is well studied for improving PLA’s mechanical and barrier properties without compromising biodegradability [24], its use to simultaneously tune electrical conductivity and melt processability in conductive PLA composites remains largely unexplored. Moreover [2], reported poor dispersion of CNC into the PLA matrix resulted in agglomeration. This can detrimentally affect composite properties by creating stress concentration points and impairing uniform conductivity. This challenge has been addressed in this study with multiple consecutive extrusion cycles.

Extrusion is a well-established scalable method that could be used to produce PLA composite filaments for FDM printing. Rheology properties are crucial for evaluating the processability and printability of these materials [21]. The melt flow behaviour governs filler dispersion, homogeneity, and filament stability during extrusion, as well as interlayer adhesion and surface finish during printing. The addition of nanofillers modifies the viscoelastic response of PLA through enhanced filler–matrix interactions and network formation, often increasing moduli and viscosity. Therefore, a detailed rheological analysis is required to clarify these structure–process–property relationships and guide the development of printable, multifunctional PLA composites.

Several studies have explored PLA composites with either carbon-based fillers [8,22,23,24,25,26] or cellulose nanocrystals [2], and a limited number have examined their combined application. Also, existing research has predominantly focused on thermal and mechanical properties, while investigations into the synergistic effects of CNC and carbon-based fillers on processability and multifunctional performance, such as electrical and thermal conductivity, remain scarce (Table 1). The key challenge remains to achieve uniform filler dispersion, strong interfacial bonding, and balanced processability and functional properties in a bio-based material suitable for smart packaging applications. In this study, PLA-based composites reinforced with CNC, GNP, and CNT were fabricated via a scalable multiple-cycle extrusion process that enables homogeneous fillers dispersion and high material throughput. The novelty of this study lies in employing this process to enhance CNC dispersion, thereby enabling uniform distribution and the formation of a three-dimensional interconnected network of hybrid GNP and CNT fillers within the PLA matrix. The central hypothesis is that the ternary system, through synergistic interactions, can achieve simultaneous improvements in electrical and thermal conductivity at reduced filler loadings, without compromising processability, compared to conventional binary systems (e.g., PLA + GNP or PLA + CNT), as summarised in Table 1. Moreover, to the best of our knowledge, this is the first study to investigate the thermal conductivity of PLA-based composites at multiple temperatures (25, 5, and −20 °C), representing typical storage and transport conditions in food and medical supply chains. This approach enables simultaneous enhancement of electrical, thermal, and rheological properties at low total filler loadings, while maintaining good melt processability. Such a combination of bio-based CNC with conductive fillers like GNP and CNT with extrusion-based additive manufacturing offers a novel, scalable pathway toward the development of high-performance PLA-based nanocomposite materials.

## 2. Materials and Methods

### 2.1. Materials

The polymer matrix used in this study was Ingeo™ Biopolymer PLA-2003D (NatureWorks LLC, Plymouth, MN, USA), with a specific gravity of 1.24 g/cm^3^ and a melt flow rate (MFR) of 6 g/10 min at 210 °C/2.16 kg.

Three nanofillers were selected for composite fabrication, including cellulose nanocrystals (CNCs, Nanografi, Ankara, Turkey) with <150 nm particle size, 10–20 nm width, and 300–900 nm length, demonstrating a density of 1.49 g/cm^3^, 92% crystallinity index, <350 µS/cm conductivity, and 4-year shelf life; graphene nanoplates (GNPs, Nanografi, Ankara, Turkey) characterised by 99.9% purity, 1.5 μm diameter, 3 nm thickness, 800 m^2^/g specific surface area, and 1500–1980 S/cm electrical conductivity; as well as industrial NC7000™ multiwall carbon nanotubes (CNTs, Nanocyl SA., Sambreville, Belgium) with 90% purity, average dimensions of 9.5 nm diameter and 1.5 μm length, transitional metal oxide below 1%, specific surface area of 250–300 m^2^/g, and volume resistivity of 10^−4^ Ω.cm.

### 2.2. Preparation of Composites

PLA composite formulations were fabricated using extrusion processing, with PLA serving as the matrix material. In addition to the neat PLA composite prepared for comparison, several composites were produced by incorporating 1% CNC along with various nanocarbon fillers (GNP and CNT). Table 2 summarises the studied compositions.

Prior to the melt blending, PLA pellets, GNP, and CNT powders were vacuum dried in an oven at 80 °C for 4 h, while the CNC powder was conditioned at 60 °C for 5 h to minimise moisture content without thermal degradation. The appropriate quantities of PLA, CNC, GNP, and CNT were manually mixed in a plastic bag and then subjected to ball milling at 80 rpm for 1 h.

Each composition mixture was melted and compounded in a twin-screw extruder Process 11 (Thermo Scientific, Waltham, MA, USA) operated at 150 rpm with torque controlled at 60–70% of system capacity. The barrel temperature profile across eight heating zones (hopper to die) was maintained at 180 °C → 190 °C → 190 °C → 190 °C → 190 °C → 180 °C → 180 °C → 175 °C. After exiting the nozzle, the extrudate was sequentially cooled through dual temperature-controlled water baths (60 °C followed by 20 °C). To achieve dispersion homogeneity, three consecutive processing cycles were implemented, each comprising extrusion, staged cooling, palletisation, and feedstock recycling for reprocessing. During this process, the filament diameter was monitored in-line using laser profilometry immediately after cooling in the water bath. Final filaments showed an average diameter of 1.70 ± 0.06 mm. All compositions and filaments were stored in desiccators containing phosphorus pentoxide to eliminate residual moisture effects.

### 2.3. Preparation of Test Samples

**Figure 1:**
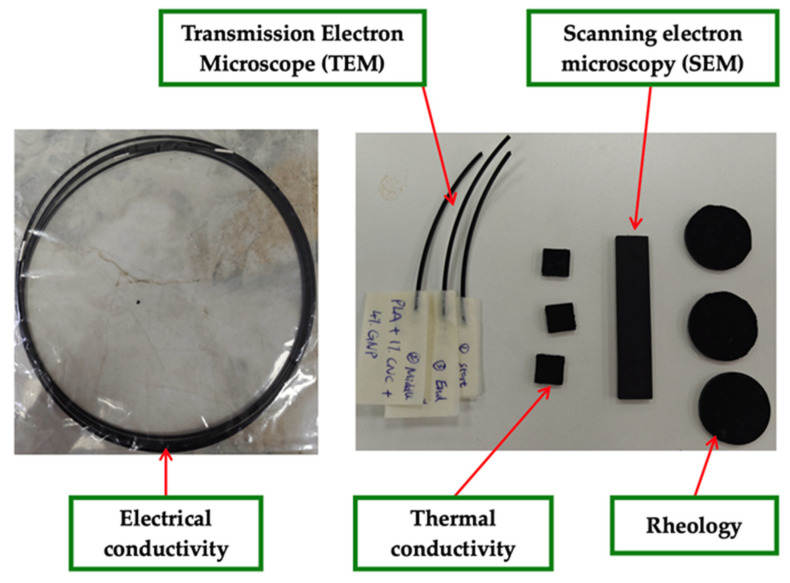
Photographic images of representative samples prepared for different characterisation techniques.

Test samples were prepared according to the specific dimensional and surface requirements of each characterisation method. Disc-shaped specimens (Ø20 mm × 1.0 mm thickness) were used for rheological measurements whereas square specimens (10 mm × 10 mm × 2.0 mm) for thermal conductivity tests. All samples were fabricated by hot-pressing PLA and PLA-based composite pellets using a Carver 3850 hot press at 180 °C under 2 tons of pressure, followed by natural cooling to room temperature to prevent thermal cracking. Electrical conductivity was evaluated separately using 1 m long filaments, and both ends were coated with 1 cm long silver paint to ensure stable ohmic contacts with the measuring electrodes. The silver coating does not affect the measurement, as it is measured the bulk conductivity. Photographs of the prepared samples are shown in Figure 1.

### 2.4. Characterisation Methods

#### 2.4.1. Scanning Electron Microscopy

Scanning electron microscopy (SEM) was conducted using a Hitachi FlexSEM 1000II SEM (Hitachi High-Tech Corporation, Tokyo, Japan) in high vacuum mode to examine the surface morphology and dispersion state of fillers within the composite matrix. Prior to imaging, samples were cryo-fractured in liquid nitrogen to expose a clean cross-sectional surface and then sputter-coated with 10 nm gold to enhance surface conductivity. SEM images were taken at 10 kV accelerating voltage at different magnifications.

#### 2.4.2. Transmission Electron Microscopy

A transmission electron microscopy (TEM) analysis was performed by Hitachi HT7800 (Hitachi High-Tech Corporation, Tokyo, Japan) at an acceleration voltage of 100 kV. Filament samples (~10 mm) were mounted in a Reichert Jung Ultramicrotome chuck, trimmed under a stereomicroscope to a ~300 µm trapezoid, and sectioned with a diamond knife into 100 nm slices. Sections were collected on 300-mesh nickel grids. The prepared samples were observed under varying magnifications to investigate their internal morphology, dispersion state, and orientation of nanofillers within the PLA matrix from the micro- to nanoscale level.

#### 2.4.3. Electrical Conductivity

**Figure 2:**
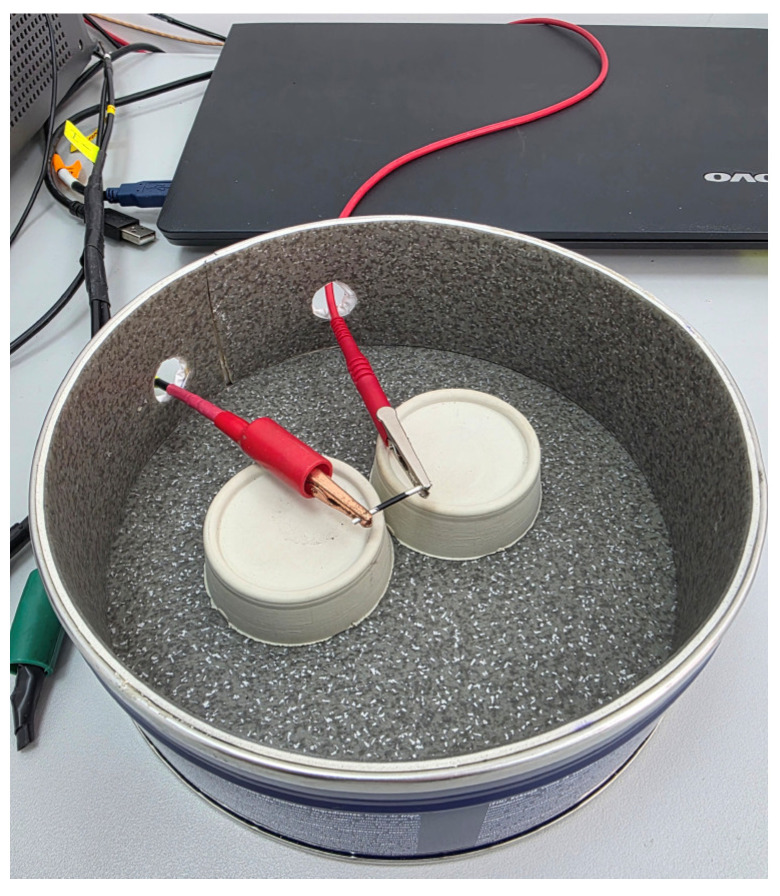
Photographic images of electrical conductivity measurement setting.

The electrical resistance was tested by the multimeter Keithley 6517B (Keithley Instruments Inc., Cleveland, OH, USA) using the R-mode test in a two-point probe configuration. To minimise external electromagnetic interference and leakage currents, all measurements were performance inside our custom-built shielding metal chamber using Triax cables (Figure 2). Samples were prepared with a fixed conductive length of 10 mm between two 10 mm silver-coated ends to minimise resistance and ensure measurable current within the instrument’s optimal range. It is worth mentioning that shorter samples length provided higher measured current at the same voltage and lower relaxation current. Therefore, the sample preparation presented in Figure 1 was modified (Figure 2). The filament diameter was measured at the centre of the filament using a precision micrometre. Due to the sample short length, diameter variation was not expected. Also, the error in diameter was maintained below ± 1%, corresponding to an approximately ± 2% error in conductivity.

The resistance of the composite samples was measured between two silver-coated ends with a fixed length of 10 mm at room temperature. Measurements were performed with a built-in input voltage of 100 V for low-conductivity materials and 2 V for high-conductive materials. For low-conductivity materials, a stabilisation period of 5 min was implemented before recording the reading to allow for current relaxation. The electrical conductivity (σ, S/m) was calculated using the following Equation (1):

(1) $$\sigma =\frac{L}{R\cdot A}$$

where L is the length between two silver-coated ends (m); R is the electrical resistance (Ω); and A is the cross-sectional area of the filament (m^2^). All measurements were performed at room temperature, and each sample was tested three times to ensure accuracy and reproducibility. The final reported conductivity is the mean of the three independent determinations. The standard deviations reported in Table 3 originate from these triplicate measurements and inherently capture sample-to-sample variations, including those arising from minor diameter fluctuations along the filament and contact quality.

#### 2.4.4. Thermal Conductivity

The thermal conductivity was evaluated using the Laser Flash Technique (LFA 467 Hyper flash, Netzsch, Hanau, Germany). Measurements were performed at 25, 5, and −20 °C, which represent common storage and transit temperatures in food and medical supply chains. Before measurements, the front and back surfaces of the samples were coated with graphite to improve their emission/absorption properties during laser heating [33]. The instrument was calibrated using a reference material (Pyroceram, Ø10 mm, thickness 2 mm) for specific heat capacity determination [33]. Thermal diffusivity and specific heat were calculated from the rear-face temperature response detected by the instrument’s infrared sensor. Sample density was obtained at room temperature via the buoyancy (Archimedes) method. Although the fundamental equation (Equation (2)) includes temperature-dependent density, a single density value was employed for all calculations. This simplification is justified for the studied PLA-based composites for two reasons. First, the experimental temperature range (−20 to 25 °C) lies substantially below the glass transition temperature of the PLA matrix (~60 °C), where the material is in a dimensionally stable, glassy state. Second, the incorporated carbon nanofillers, which possess very low or even negative thermal expansion coefficients, form a rigid, interconnected network that mechanically constrains the thermal expansion of the polymer matrix. This results in negligible volumetric change across the tested interval, thereby ensuring the consistency and comparability of the thermal conductivity results calculated with a single density value for all temperature points.

Thermal conductivity (λ, W/mK) was calculated using the following equation:

(2) $$\lambda \left(T\right)=\alpha (T)\cdot C_{p}(T)\cdot \rho (T)$$

where α is the thermal diffusivity (m^2^·s^−1^), $C_{p}$ is the specific heat capacity (J·kg^−1^·K^−1^), and ρ is the bulk density (kg·m^−3^). All measurements were performed three times and reported as average results. Data analysis and calculations of the thermal conductivity were carried out using Proteus^®^ Professional software (Version 8.6, Netzsch).

#### 2.4.5. Rheological Measurements

Rheological properties were carried out using a stress-controlled rheometer (AR-G2, TA Instruments, New Castle, DE, USA) equipped with an electrically heated parallel plate (EHP) geometry. The oscillatory tests were performed at a temperature of 190 °C using plates with a 25 mm diameter and 1000 microns fixed gap. A strain (amplitude) sweep test was initially performed at 1 Hz over a strain range from 0.01 to 1000% to identify the linear viscoelastic region (LVR) for each sample [34]. The end of the LVR was identified from the graph of log G’ versus log γ.

The cohesive energy density was calculated from the amplitude sweep data to evaluate the internal strength of the filler–polymer network and the level of interfacial compatibility [35,36]. It represents the energy a material can store elastically before yielding or internal failure. Cohesive energy density ($E_{c}$, J/m^3^) was calculated using the following equation [35]:

(3) $$E_{c}=\frac{1}{2}G_{c}^{\prime }\gamma_{c}^{2}$$

where $G_{c}^{\prime }$ is the average storage modulus within the LVR (Pa), and $\gamma_{c}$ is the critical strain (dimensionless).

The yield stress was determined from the amplitude sweep results by plotting the elastic stress as a function of absolute strain, where elastic stress (τ, Pa) was calculated as:

(4) $$\tau =G^{\prime }\times \gamma$$

where $G^{\prime }$ is storage modulus (Pa), and γ is the absolute strain (dimensionless).

The maximum point on this curve was taken as the yield stress and corresponding strain as the yield strain, representing the onset of structural breakdown or the transition from solid-like to flow behaviour.

After the LVR was established, frequency sweep tests were conducted at 190 °C with the linear region to examine the viscoelastic behaviour of the samples. The tests covered an angular frequency range of 0.1–100 rad/s. For neat PLA and composites containing 0.5 wt% carbon nanofillers, a constant strain of 1% was used. In contrast, for composites with higher nanofiller loadings, the strain was reduced to 0.5% to ensure the measurements remained within the LVR and to maintain data accuracy.

The storage modulus (G′, Pa) and loss modulus (G″, Pa) were recorded as functions of angular frequency (ω) from the frequency sweep data. Complex viscosity (|η*|, Pa·s), which reflects the material’s overall resistance to oscillatory deformation, was calculated as:

(5) $$\left|\eta *\right|=\frac{\sqrt{{G\prime }^{2}+G^{\prime \prime 2}}}{\omega }$$

### 2.5. Prototyping Using Fused Deposition Modelling (FDM) 3D Printing

Prototype boxes and lids were produced through FDM 3D printing using PLA-based nanocomposite filaments produced as described in Section 2.2. The box, with dimensions of 35 mm × 60 mm × 17 mm, and 2 mm thickness were modelled and then 3D printed using the PLA, PLA/1% CNC/4% GNP, and PLA/1% CNC/2% GNP/2% CNT filaments. The lid, with dimensions of 35 mm × 60 mm and 4 mm thickness, was also printed using the same filaments. The 3D printing was performed using a fused deposition modelling (FDM/FFF) 3D printer (X400 PRO, German RepRap, Feldkirchen, Germany) equipped with an extrusion nozzle of 0.4 mm diameter. The printing parameters were set to a nozzle temperature of 200 °C, an extrusion speed of 50 mm/s, and the heated platform temperature of 65 °C. Samples were printed with a layer height of 0.2 mm and 100% infill, with each layer deposited in a rectangular direction of one layer to another.

## 3. Results and Discussion

### 3.1. Production of PLA and PLA-Based Composite Filaments

**Figure 3:**
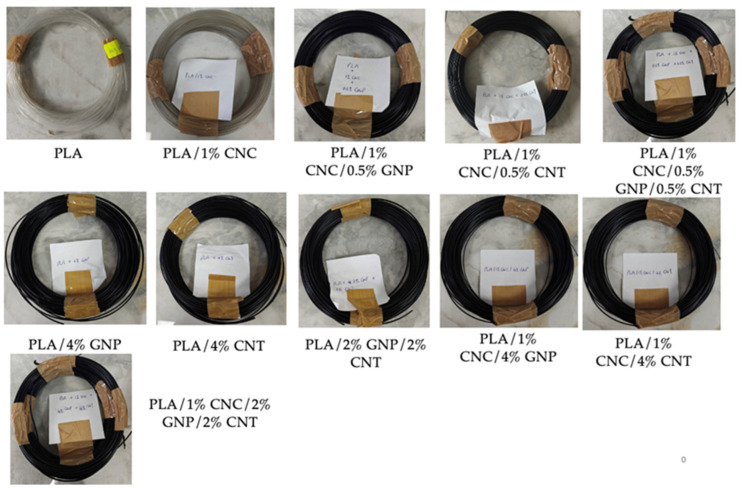
Visual appearance of 11 composite filaments including neat PLA and PLA-based composites containing various nanofillers and concentrations.

Eleven extruded PLA and PLA composites, containing the three different nanofillers CNC, GNP, and CNT individually and in combination, were prepared to produce mono-, bi-, and tri-filler systems of PLA-based filaments (Figure 3), suitable for 3D printing using the same extrusion conditions. This approach enabled a systematic investigation of the effects of nanofiller type and concentration on the morphology, electrical and thermal conductivity, and rheological behaviour of the resulted PLA-based composites. The produced filaments showed uniform surfaces with no visible defects (e.g., voids or irregularities). All filaments maintained an average diameter of approximately 1.70 mm, as quantified by laser profilometry, confirming stable extrusion conditions during processing (Figure 3). The neat PLA filament exhibited high transparency, whereas the addition of CNC caused a slight loss of transparency with a faint grey appearance, probably due to light scattering from dispersed nanocrystals [37]. In contrast, the incorporation of carbonaceous nanofillers (GNP and CNT) produced black filaments explained by the intrinsic colour and uniform distribution of the carbon fillers within the matrix [37]. At higher carbon filler concentrations, the filament surfaces appeared slightly rougher than neat PLA, likely due to the carbon particles becoming exposed at the filament surface.

### 3.2. Structure and Morphology

A uniform dispersion of nanofillers within the PLA matrix is essential for obtaining consistent electrical, thermal, and rheological performance [24]. SEM and TEM were used to evaluate the microstructure and filler distribution.

**Figure 4:**
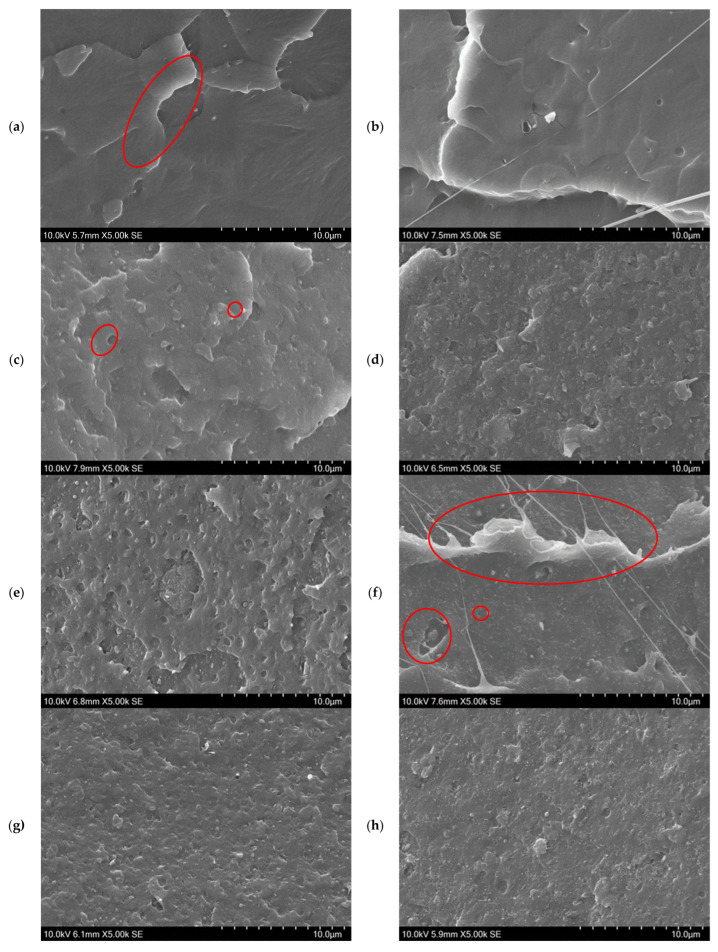
SEM micrographs of the cryo-fractured surfaces of (**a**) neat PLA, (**b**) PLA/1% CNC, (**c**) PLA/1% CNC/0.5% GNP/0.5% CNT, (**d**) PLA/1% CNC/2% GNP/2% CNT, (**e**) PLA/4% GNP, (**f**) PLA/4% CNT, (**g**) PLA/1% CNC/4% GNP, and (**h**) PLA/1% CNC/4% CNT. Red circle in (**a**), (**c**), and (**f**) highlight sharp fracture lines, micro-voids, and a fibrous texture with elongated ridges, respectively.

Figure 4 presents SEM micrographs of the cryo-fractured surfaces of neat PLA and selected nanocomposites containing CNC, CNT, GNP, and their hybrids. All images are at the same magnification of 5000×, for easy comparison. The neat PLA (Figure 4a) exhibits a smooth and featureless fracture surface with sharp fracture lines, confirming a homogeneous polymer matrix. The incorporation of CNC (Figure 4b) introduces fine microcracks and shallow depressions, indicating that CNC particles were embedded within the PLA matrix and contributed to slightly rougher surface. Similar roughening has been reported for the PLA/1%CNC system attributed to strong interfacial hydrogen bonding between PLA and the hydroxyl groups on CNC [2,38].

At 0.5 wt% carbon hybrid composite (Figure 4c), the surface becomes noticeably rougher with the appearance of micro-voids and irregular features, suggesting the successful incorporation of nanofillers and filler–matrix interactions. A significant morphological transformation occurs at high hybrid filler loading (Figure 4d), where the surface exhibits increased surface texture with interconnected features [24]. This structure suggests the development of a percolated conductive network through filler–filler contacts. While the surface irregularities indicate some degree of filler agglomeration, the presence of interconnected pathways is favourable for conductive network development [29]. The extent of filler–matrix adhesion and stress transfer efficiency would depend on the balance between filler networking and dispersion quality. Overall, the progressive surface roughening with increasing filler content confirms that the nanofillers are successfully incorporated and dispersed within the PLA matrix, leading to enhanced morphological stability [24]. The morphology of the high-carbon composites (Figure 4e,f) highlights the influence of filler type and the role of CNC addition. Figure 4e shows a layered and plate-like fracture pattern, typical of GNP stacking, while those containing only CNT (Figure 4f) show a dense, fibrous texture with elongated ridges. Both carbon-rich systems demonstrate well-formed networks but display local surface irregularities and small voids, suggesting limited matrix wetting at high filler concentrations. When CNC is incorporated into these high-carbon systems (Figure 4g,h), the surfaces become more homogeneous, compact, and continuous. The GNP-based composite with CNC (Figure 4g) shows fewer gaps between plate-like structures, while the CNT-based composite with CNC (Figure 4h) exhibits smoother and more cohesive fracture features. These morphological improvements demonstrate enhanced dispersion and more effective integration of carbon fillers within the PLA matrix, suggesting that CNC functions as a bridging and stabilising agent. The addition of CNC therefore promotes a more uniform and integrated structure, reducing aggregation, and facilitates network continuity and connectivity, which are also critical for achieving a lower percolation threshold and improved conductive pathways. This refined morphology supports the enhanced electrical, thermal, and changes in the rheological properties discussed later in sections.

**Figure 5:**
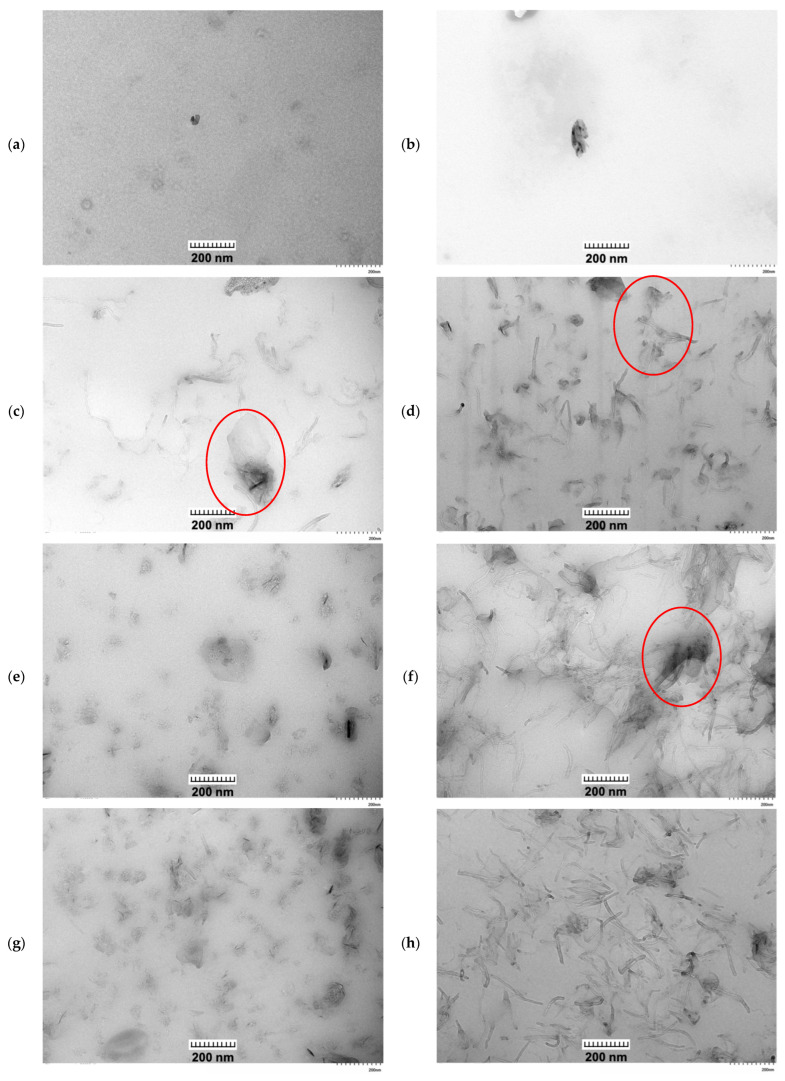
TEM images of (**a**) neat PLA, (**b**) PLA/1% CNC, (**c**) PLA/1% CNC/0.5% GNP/0.5% CNT, (**d**) PLA/1% CNC/2% GNP/2% CNT, (**e**) PLA/4% GNP, (**f**) PLA/4% CNT, (**g**) PLA/1% CNC/4% GNP, and (**h**) PLA/1% CNC/4% CNT. All images were acquired at 40,000× magnification with a scale bar of 200 nm for comparison. Red circles in (**c**,**d**) highlight CNTs bridging between GNP sheets, while red circle in (**f**) indicates an agglomerated CNT bundle.

While SEM provided insight into the surface morphology of the composites, TEM was further employed to examine the nanofiller dispersion, interfacial adhesion, and network formation of CNC, CNT, and GNP within the PLA matrix. TEM images (Figure 5) show distinct morphological differences among the mono-, bi-, and tri-filler systems, consistent with the SEM observations discussed earlier. The neat PLA (Figure 5a) shows a mostly homogeneous and featureless matrix, as per the SEM image. On the other hand, small rod-like CNC particles in PLA/1%CNC appear well dispersed without significant aggregation, indicating effective filler–matrix interactions that suppress CNC self-agglomeration during processing. In mono-filler systems (Figure 5e,f), a clear difference in morphology is observed. The dispersion state and filler distribution strongly depend on the type of filler. For the PLA/GNP composite (Figure 5e), GNPs are randomly distributed within the matrix but remain isolated, consistent with the platelet-like geometry that promotes planar stacking rather than long-range connectivity [26,39]. Conversely, the PLA/CNT composite (Figure 5f) exhibited entangled and partially agglomerated bundles of CNT, forming localised conductive paths [26,39,40]. When CNC is incorporated (bi-filler composites, Figure 5g,h), GNP or CNT filler becomes more homogeneous dispersion, and the interfaces appear smoother. The GNP appears more evenly distributed with fewer voids, while CNT bundles are finer and more uniformly dispersed. The presence of CNC effectively reduces the degree of agglomeration and enhances the homogeneity of the nanofiller dispersion. This observation suggests that CNC functions as a stabilising interfacial modifier, enhancing filler–matrix compatibility and promoting uniform dispersion of carbon fillers within the PLA matrix. In the tri-filler composites (Figure 5c,d), CNC, CNT, and GNP are clearly visible within the PLA matrix, forming a complex, multiscale architecture. CNTs often bridge between GNPs sheets, generating a hierarchical, three-dimensional network that enhances the continuity of the percolating network, confirming synergistic interactions among nanofillers [23,26]. At the low loading (PLA/1% CNC/0.5% GNP/0.5% CNT, Figure 5c), these fillers were dispersed, but most of them stayed isolated, suggesting uniform dispersion and early-stage network formation. With increasing carbon content (PLA/1% CNC/2% GNP/2% CNT, Figure 5d), denser regions with entangled CNT bundles and overlapping GNP sheets appear, indicating strong filler–filler contact and a partial network. Although some localised agglomerates are evident, most carbon domains remain interconnected within the polymer matrix, forming partially connected conductive paths [23,26].

Overall, the morphological evolution across the mono-, bi-, and tri-filler systems demonstrates an enhancement of filler dispersion, interfacial adhesion, and the formation of interconnected networks within the PLA matrix, consistent with previous studies of hybrid carbon nanocomposites [23,24,26,39]. The incorporation of CNC further improves filler dispersion, while the synergistic interaction between GNP and CNT enables the formation of hierarchical networks capable of improving the electrical conduction and thermal transport within the PLA matrix, as well as altering the system rheological behaviour.

### 3.3. Electrical Conductivity

The electrical conductivity of neat PLA and its composites is summarised in Table 3. The conductivities of neat PLA and most composites were below the detection limit of the measurement equipment.

Neat PLA typically exhibits very low conductivity (~$10^{-13}-10^{-10}$ S/m) [23], consistent with its inherently insulating nature. The inability of the equipment to detect these values confirms the conductivity of neat PLA, and PLA/1% CNC composites remain below the measurable range. Electrical conductivity in polymer nanocomposites generally increases with filler loading until the percolation threshold is reached, beyond which a continuous conductive network forms and conductivity rises sharply [23]. Although the incorporation of 0.5 wt% carbon fillers led to a measurable increase in electrical conductivity, the resulting values ($\sim 10^{-11}$ S/m) remain characteristic of an insulating, sub-percolative regime. At this low filler content, conductive domains remain isolated within the PLA matrix without forming a continuous network, as confirmed by TEM images (Figure 5a–c).

A significant increase in conductivity was observed only in the CNT-containing systems. The PLA/4% CNT composite exhibited a conductivity of 30.70 S/m, demonstrating the formation of a continuous and efficient conductive network at relatively low CNT loading. This is almost an order of magnitude higher than the 0.021 S/m for PLA/6% CNT [23] and 4.54 S/m reported for PLA/12% CNT systems [25], indicating excellent CNT dispersion and interconnection within the PLA matrix after three consecutive processing cycles. The sharp increase in conductivity between 0.5 wt% and 4 wt% CNT indicates that the electrical percolation threshold occurs within this range, beyond which a connected network enables long-range electron transport. In contrast, GNP-based composites remained below the detection limit at 4 wt%, confirming that percolation was not achieved, as evidence by isolated nanofillers within the matrix in TEM images. This observation aligns with previous studies reporting that PLA/GNP systems require 7–10 wt% GNP to reach the percolation threshold, yielding conductivities around 4–6 S/m [29,41,42,43,44]. However, these values are not universal and are strongly dependent on the graphene type/grade (e.g., flake size, aspect ratio, surface area, oxidation level, and defect density), which significantly impacts the effectiveness of network formation and therefore the required percolation loading. CNTs, with their high aspect ratio (>1000) and one-dimensional geometry, form long-range entangled networks that facilitate electron tunnelling between adjacent nanotubes and generate a three-dimensional conductive network (Figure 5f) [23,26]. In comparison, GNP has a two-dimensional lamellar structure, requiring extensive face-to-face contact to achieve an effective conductive path (Figure 5e).

The hybrid composites containing both GNP and CNT exhibit intermediate electrical conductivities between those of the mono-filler systems. PLA/2% GNP/2% CNT composite achieved an electrical conductivity of 0.16 S/m, which is 99.48% lower than that of the PLA/4% CNT system (30.70 S/m) and about 300 billion percent larger than that of PLA/4% GNPcomposite (5.30 × 10^−11^). Although there were some improvements in the electrical conductivity in the systems with CNC, the same trend was observed, indicating that CNT is the main contributor to the electrical conductivity of PLA with GNP/CNT filler. Indeed PLA/1% CNC/4% CNT has an electrical conductivity of 38.30 S/m, which is far higher than that of PLA/1% CNC/2% GNP/2% CNT (1.27 S/m). TEM images (Figure 5e,f) reveal that CNTs often wrap around or connect GNP edges, forming partial conductive bridges. This configuration results in a three-dimensional hybrid network with limited continuity, which accounts for the lower conductivity observed.

As mentioned before, the addition of 1 wt% CNC improved the electrical conductivity of PLA-based composites. In the hybrid system (PLA/1% CNC/2% GNP/2% CNT), CNC incorporation produced a 693.75% increase in conductivity relative to its CNC-free hybrid system. A smaller improvement was observed in the CNT-containing composite, where PLA/1% CNC/4% CNT showed a 24.76% increase compared with PLA/4%CNT. This enhancement is primarily attributed to the ability of CNC to promote carbon filler dispersion and conductive network formation. This enhancement highlights the crucial role of CNC as a dispersion aid and conductive network regulator. CNC introduces abundant surface hydroxyl groups that could participate in hydrogen-bond-assisted interactions with the ester carbonyl groups of PLA and oxygen-containing functional groups on CNT and GNP surfaces. These interactions suppress carbon filler agglomeration during melt mixing and promote a more uniform carbon filler distribution. This improved dispersion reduces interparticle distance, thereby lowering tunnelling resistance and enhancing charge transport efficiency. In addition, CNC acts as a structural spacer, preventing re-stacking of GNP sheets and encouraging CNT to bridge across adjacent GNP layers. The resulting three-dimensional hybrid network ensures that electron transport can proceed through both tunnelling and direct contact conduction pathways.

Overall, these results demonstrate that CNTs dominate the electrical transport behaviour due to their high aspect ratio and effective network formation. Although CNC-GNP-CNT systems exhibit lower conductivity than CNT-only systems, CNC-assisted hybridisation improves filler dispersion and network stability at reduced CNT contents. Moreover, the achieved electrical conductivity, though lower than that of metal-based conductors, falls well within the range required for several smart packaging and sensing functionalities. In practical packaging systems, conductivities in the range of $10^{-3}-10^{1}$ S/m are commonly sufficient for antistatic charge dissipation, resistive or impedance-based sensing, and signal transduction layers in printed sensor architectures [45,46]. The present electrical conductivity enables the formation of continuous conductive pathways capable of responding to mechanical deformation, environmental stimuli, or interfacial changes, which are central to strain, pressure, and chemical sensing concepts [46,47,48]. This conductivity was achieved at relatively low filler content, preserving the mechanical compliance, processability, and scalability required for flexible and disposable packaging platforms, where lightweight, low-cost, and environmental materials may be prioritised over metal-like conductivity.

### 3.4. Thermal Conductivity

**Figure 6:**
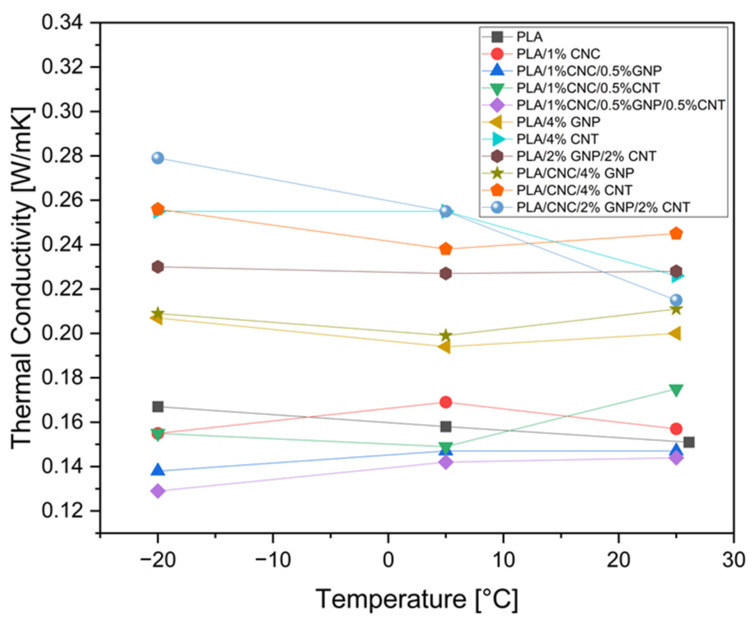
Thermal conductivity of PLA-based composites at 25, 5, and −20 °C. Data points are average of 3 replicates.

Thermal conductivity defines the ability of a material to transfer heat. It is a critical parameter for smart packaging and electronic applications, where precise thermal management is required to ensure accurate temperature monitoring and stable device performance. In this study, thermal conductivity measurements were performed at –20 °C, 5 °C, and 25 °C to evaluate the heat transfer efficiency of the composites under typical storage and operational conditions relevant to food and medical applications (Figure 6).

Heat transport in polymer-based materials is a complex process and influenced by multiple factors, including temperature, crystallinity, molecular chain orientation, and interfacial thermal resistance [49,50,51,52]. Indeed, due to the absence of free charge carriers in polymers, phonons act as the dominant heat carriers, and their transport is strongly limited by molecular disorder and thermal resistance at filler–matrix and filler–filler interfaces. Unlike electrical conduction, which requires the formation of a fully percolated conductive network, thermal conductivity in polymer composites is predominantly governed by phonon-mediated heat transfer and is less sensitive to strict network continuity.

As expected, neat PLA exhibited low thermal conductivity values ranging from 0.151 to 0.167 W/mK across the investigated temperature range. The obtained data reflect the limited phonon transport capability of the amorphous polymer matrix [24]. The introduction of 1 wt% CNC resulted in only a minor increase, confirming that CNCs act primarily as dispersion aids and interfacial modifiers rather than effective heat-transfer agents. Composites containing 0.5 wt% carbon nanofillers and their hybrid exhibited thermal conductivities comparable to or lower than that of neat PLA. This suggests that isolated fillers at such low concentrations are insufficient to establish continuous phonon transport pathways and may instead introduce additional phonon-scattering interfaces [53].

A progressive enhancement of thermal conductivity was observed with increasing nanofiller content. Mono-filler composites containing 4 wt% GNP or CNT display higher thermal conductivities than neat PLA, with CNT-based systems outperforming GNP ones. This demonstrates that CNTs are the primary contributors to thermal transport in the PLA matrix due to the formation of continuous, entangled CNT networks that enable efficient phonon transport. In contrast, the GNP-filled composites exhibited comparatively lower thermal conductivities, which can be associated with isolated and poorly connected platelets, preventing the formation of efficient in-plane heat-transfer channels (Figure 5e–h). These results differ from the results of Ivanov et al. (2019) and Spinelli et al. (2019), where GNP-based composites outperform CNT ones at the comparable loading because well-dispersed GNPs provided broad interfacial contact areas that promoted in-plane conduction [23,25]. The results underscore the strong dependence of thermal transport on graphene type, size, dispersion quality, and interfacial interactions within the polymer matrix.

For hybrid systems like PLA/2% GNP/2% CNT composite, the incorporation of both 1D CNT and 2D GNP fillers create 3D thermal pathways, which reduces inter-filler thermal resistance and facilitates phonon transfer across multiple length scales [24,25]. However, this 3D thermal pathway does not lead to a universal synergistic enhancement in thermal conductivity compared to PLA/4% CNT probably. This finding collaborates with the literature reporting that the CNT/GNP synergistic effect in polymers matrix is strongly composition dependent, requiring optimisation of the filler ratio to avoid anti-synergistic behaviour through partial disruption of CNT, the entangled network by GNP platelets, and reduction of its continuity [54]. At 25 °C, the thermal conductivity of PLA/2% GNP/2% CNT is comparable to PLA/4% CNT, while at 5 and −20 °C, it remains slightly lower. Notably, a temperature-dependent synergistic effect becomes more pronounced when CNC is introduced into the hybrid system. PLA/2% GNP/2% CNT composite shows an increase in thermal conductivity of 12.3% at 5 °C and 21.3% at –20 °C relative to the CNC-free hybrid, and an overall enhancement of approximately 68% compared with neat PLA at –20 °C. Under sub-ambient conditions, reduced phonon–phonon scattering amplifies the influence of interfacial thermal resistance, thereby increasing the effectiveness of CNC-assisted dispersion and improved filler–matrix contact phonon transfer [53].

In general, thermal conductivity exhibited a tendency to increase with decreasing temperature, which can be attributed to decreased phonon–phonon scattering that improves interfacial contact at lower thermal energy levels. This temperature dependence became less pronounced at higher nanofiller loadings, where heat transport was increasingly governed by the dense and continuity of carbon-based networks.

The improvement of thermal conductivity in these composites can be closely related to the quality of the filler–matrix interfaces and the organisation of the filler networks. Improving interfacial contact and more continuous filler pathways enhance phonon coupling and suppress interfacial scattering, leading to higher conductivity, whereas poorly distributed fillers introduce additional scattering sites that impeded thermal transport. In our composites, CNC contributes to this effect by improving fillers dispersion and enhancing network architecture, thus reducing thermal boundary resistance under favourable conditions. Nevertheless, both PLA and carbon nanofillers contain intrinsic structural defects that act as phonon-scattering centres. As a result, the measured thermal conductivities remain well below the theoretical values of the fillers themselves. The limited improvement observed for mono-filler CNT systems (67%) reflects the inherently anisotropic geometry of the nanotubes, which forms efficient percolating pathways compared with the planar overlap achievable by GNPs.

Overall, the observed results indicate that thermal conductivity enhancement in PLA-based composites is primarily driven by CNT networks, while hybridisation of CNT with GNP and CNC provides a secondary and strongly temperature-dependent contribution. The observed synergistic effects are limited in magnitude and become significant only under sub-ambient conditions where interfacial transport mechanisms dominate phonon propagation.

### 3.5. Rheological Properties

Three consecutive extrusion cycles were employed to ensure adequate melt homogenisation and uniform dispersion of fillers within the PLA matrix. Acknowledging that repeated extrusion of PLA can potentially induce polymer degradation and alter its properties, the thermal and rheological behaviour of both the neat polymer and the processed material were evaluated. Based on the thermal data (Table 4), the three-cycle processing approach resulted in only minor changes in thermal properties. Indeed, the degradation onset temperature remained essentially unchanged after extrusion (341.10 °C for virgin PLA and 341.34 °C for PLA after three extrusion cycles, whereas only a slight decrease in glass transition temperature (from 58.72 °C to 57.48 °C) and melt temperature (from 154.08 °C to 150.94 °C) were observed before and after processing). These findings suggest limited chain scission and enhanced chain mobility induced by repeated melt processing, consistent with results from previously reported reprocessed PLA systems [55]. Importantly, all thermal parameters remained within the typical ranges reported for Ingeo 2003D PLA.

Similarly, three extrusion cycles induced minor changes in the rheological properties of PLA (Table 4). Extruded PLA exhibited a 10.59% reduction in yield stress compared to virgin PLA, indicating a slight softening of the material, probably due to molecular weight loss from chain scission during melt extrusion [56]. The yield strain increased (2.36 vs. 2.07), reflecting improved flexibility and ductility in the extruded PLA, which can be attributed to enhanced chain mobility after extrusion. The critical strain was nearly identical for both materials, demonstrating that extrusion did not significantly affect the material’s deformation capacity. However, $E_{c}$ of extruded PLA decreased by 20.8% compared to virgin PLA, indicating reduced intermolecular interactions, possibly due to structural changes during extrusion. These thermal and rheological results suggest that the three extrusion cycles induced only mild material modification, with no significant impact on processing stability (e.g., melt flow rate, processing temperature). Therefore, the applied reprocessing strategy ensures material homogeneity without significantly altering the material’s thermal or flow properties [51].

**Figure 7:**
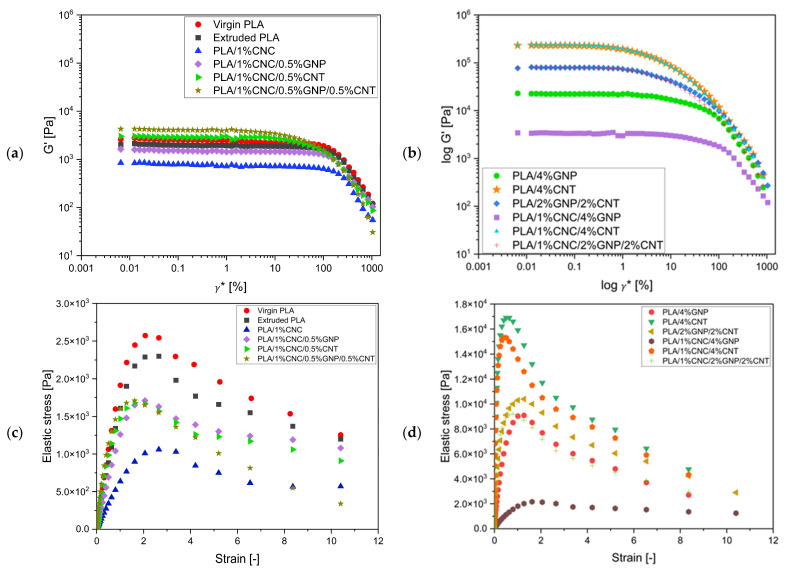
Amplitude sweep tests of PLA-based composites: (**a**,**b**) log–log plots of storage (G′) versus strain (γ *) showing the linear viscoelastic region, and (**c**,**d**) yield stress behaviours at low and high filler concentrations, respectively. Data points are average of 3 replicates (*n* = 3).

In 3D printing, the filament is extruded through a heated nozzle and experiences dynamic shear forces, highlighting the importance of studying its rheological properties. The rheological behaviour of PLA and the produced composites were investigated using dynamic oscillatory rheology, which combines amplitude and frequency sweep tests to examine their melt structure and energy dissipation mechanisms. Amplitude sweep measurements (Figure 7) allow the determination of the linear viscoelastic region (LVR) and to extract the yield stress, yield strain, and critical strain (Table 3), describing the onset of network breakdown. The corresponding cohesive energy density ($E_{c}$), derived from amplitude data, quantifies the energy a material can store elastically before yielding or internal failure [35]. Frequency sweep tests assess the material’s viscoelastic response, where the storage modulus (G′) and loss modulus (G″) (Figure 7a,b) represent its elastic and viscous components, respectively, and the complex viscosity (|η*|) (Figure 7c,d) reflects the resistance to deformation across shear frequencies. Together, these parameters collectively provide critical insights into the melt viscoelasticity, filler–matrix interactions, and structural integrity that govern processability and end-use performance, particularly in melt processing such as extrusion and FDM printing. Based on these rheological indicators, the formulations show potential for suitability in FDM applications.

**Figure 8:**
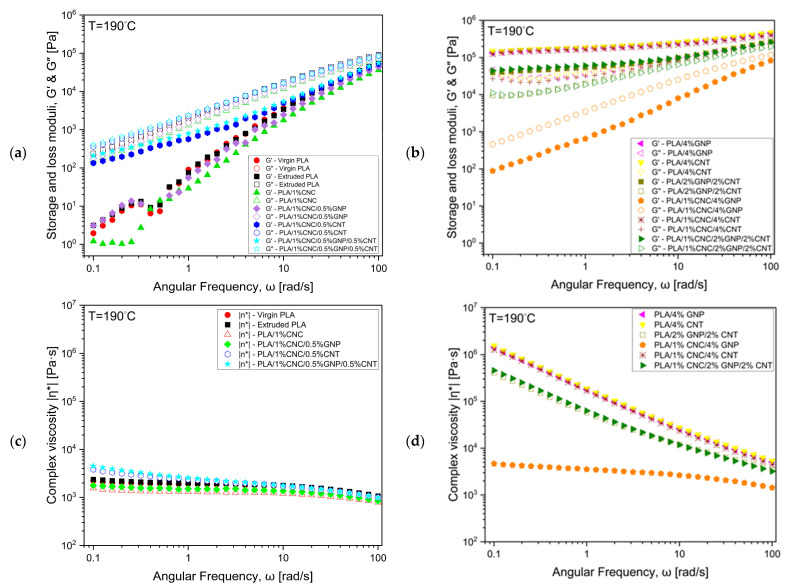
Frequency sweep tests of PLA-based composites: storage (G′) and loss (G′′) moduli at (**a**) low and (**b**) high filler concentrations, and complex viscosity (|η*|) at (**c**) low and (**d**) high filler concentrations as functions of angular frequency (ω) at 190 °C. Data points are average of 3 replicates (*n* = 3).

The rheological response of neat PLA and PLA/1%CNC composites forms the fundamental viscoelastic baseline for evaluating nanofiller effects. Neat PLA displayed typical viscoelastic behaviour, characterised by a broad LVR and low G′, G″, and |η*|, along with the highest $E_{c}$ (487.05 J/m^3^). This reflects a cohesive, well-entangled polymer melt capable of storing and dissipating deformation energy effectively [57]. Incorporation of 1 wt% CNC reduced G′, G″, |η*|, and $E_{c}$ (238.58 J/m^3^), demonstrating a 22.4% decrease in E_c_ (Figure 7 and Figure 8, Table 5). Since $E_{c}$ quantifies the strength of polymer–polymer interactions [52], this reduction in $E_{c}$ and rheological properties can be attributed to the disruption of intermolecular interactions (van der Waals, hydrogen bonding) between PLA chains through multiple possible mechanisms [52,56,58,59]. These include CNC acting as a nano-spacer that mitigates the carbon fillers re-agglomeration network [21,22,23], but also physically separate PLA chains, and disrupt their native interactions. Moreover, CNCs may displace direct PLA-PLA contacts, thereby facilitating the formation of PLA-CNC-PLA interfacial interactions via hydrogen bonding, which have been demonstrated to be weaker [58]. In fact, ref. [58] found that PLA- chitin or PAL- cellulose interactions via polysaccharide hydrogen bonds are of lower strength than PLA–PLA interactions, which may have contributed to the reduced net interaction energy between polymer chains. It has also been reported that polymers near filler surfaces are often elongated and flattened, with modified local dynamics with faster relaxation when polymers with filler interactions are weak, or slower when they are strong [59]. A structural rearrangement disrupting the dense packing necessary for strong interchain cohesion, and therefore, reducing the cohesive energy, could be hypothesised. On the other hand, the critical strain increased from 63.34% to 79.59%, showing a broader LVR in the PLA/1% CNC composite. Overall, PLA/1% CNC composite became more fluid-like (G″ > G′), which could be associated with an improved flowability with lower melt strength and shape retention after deposition.

The incorporations of carbon nanofillers significantly modify the viscoelastic response of PLA matrix, shifted the viscoelastic profile toward solid-like behaviour, indicating the formation of progressively interconnected networks. The addition of GNP increased G′, G″, |η*|, accompanied by a moderate reduction in LVR (Figure 7 and Figure 8). The composite remained a stronger elastic response while maintaining viscous-dominant (G″ > G′), indicating the filler network remained below full percolation [60]. This improvement stems from GNP’s high aspect ratio and 2D planar geometry, which restrict polymer-chain mobility. Yield stress increased while the yield strain decreased, reflecting the formation of a stiffer but slightly brittle structure. π–π stacking and van der Waals interactions between PLA’s aromatic backbone and GNP surfaces, assisted by CNC-mediated dispersion through hydrogen bonding, promoted a well-dispersed microstructure that allows energy storage and dissipation under strain. The relatively high $E_{c}$ (≈297 J/m^3^) indicates the system maintains strong toughness and internal cohesion.

In contrast, CNT addition produced a sharper rise in G′, G″, and |η*| than the GNP-reinforced system at equivalent loadings (Figure 7 and Figure 8), indicating stronger elastic energy storage and higher melt stiffness [39]. The viscoelastic enhancement arises from high aspect ratio and the formation of a semi-connected CNT filler framework that limits polymer chain mobility and restricts molecular relaxation [60]. However, the narrower LVR implies that CNT-reinforced melts are stiffer but less tolerant to strain, with earlier structural breakdown under the tested conditions. The $E_{c}$ drastically dropped to 9.2 J/m^3^ (Table 4), showing that the polymer–polymer chains interaction within the melt significantly decreased. This behaviour stems from rigid CNT bundles that act as localised stress concentration, promoting brittle fracture rather than distributed energy absorption. Consequently, CNT reinforcement produces a stiffer but less cohesive melt, combining high moduli with low ductility.

Hybrid GNP-CNT composites demonstrated increased G′, G″, |η*|, and shear-thinning relative to individual fillers at low loadings, but the enhancement became less pronounced at higher concentrations, indicating synergistic filler networking [61]. The moderation at high loading is attributed to filler–filler interaction where coexisting nanofillers hinder percolation, leading to limited network connectivity [62]. The viscous-dominant regime suggests that complete percolation had not yet occurred, but a semi-connected network was established. The intermediate $E_{c}$ (5.20–12.74 J/m^3^) indicates a balance between the stiff-toughness profile. Overall, hybrid systems offer synergistic reinforcement, achieves superior rheological stability and melt integrity, while maintaining acceptable processability [39,62].

The introduction of CNC into PLA-carbon filler composites significantly alters their rheological properties by regulating rigid carbon filler dispersion and networks. CNC interferes with these percolated, rigid filler networks, resulting in a more homogeneous structure. This results in slightly lower G′, |η*| and $E_{c}$ compared with their CNC-free equivalents. $E_{c}$ is critical metrics for processability, as it determines the structural failure point. PLA/4% CNT exhibited a very low $\gamma_{c}$ with a very high ${G_{c}}^{\prime }$, a direct theological predictor of high extrusion pressure and nozzle clogging. The addition of 1% CNC precipitates a drastic 75% reduction in $E_{c}$, accompanied by a 50.96% decrease in critical strain. It shifts in failure mode from catastrophic network facture to early, localised yielding at weaker junctions, thereby facilitating melt flow and eliminating processing bottlenecks. For the GNP-based system, CNC addition reduces $E_{c}$ by 25% but increases critical strain by 121.33%, which reduces ${G_{c}}^{\prime }$ by 85%, indicating a transition to a more ductile melt capable of substantial deformation before yielding, which is highly beneficial for filament spreading and inter-layer adhesion during printing. In hybrid systems, such as PLA/2% GNP/2% CNT, CNC bridges the mismatched carbon fillers, enhancing $E_{c}$ by 21.35%. This change in rheological properties might initially appear to contradict the observed improvement in conductivity. However, this can be explained by the distinct mechanisms governing each property. Electrical conductivity is primarily determined by the quantity and connectivity of filler contacts, which are maximised by uniform filler dispersion. In contrast, rheological properties like ${G_{c}}^{\prime }$ and $E_{c}$ are dominated by the strength of the strong filler–filler junctions and the rigidity of filler agglomerates. CNC disrupting these rigid agglomerates and replacing strong carbon–carbon contacts with softer CNC-mediate interfaces resulted in a more processable pseudo-solid melt. This disruption improves printability by preventing nozzle clogging during 3D printing, despite the decrease in material rigidity.

Raising the total carbon filler content from 0.5 to 4 wt% significantly increased G′, G″, and |η*| (Figure 8), confirming the transition of PLA-based melts from viscous-dominant to elastic and solid-like behaviour as interconnected filler–polymer networks formed and restricted molecular relaxation [60]. The LVR narrowed and $E_{c}$ lowered, indicating earlier structural breakdown and reduced strain tolerance, a typical sign of increased brittleness. The stiffness–toughness trade-off becomes apparent as denser filler networks are formed, causing more deformation energy to be stored elastically rather than dissipated through chain relaxation, yielding higher moduli but lower $E_{c}$.

**Figure 9:**
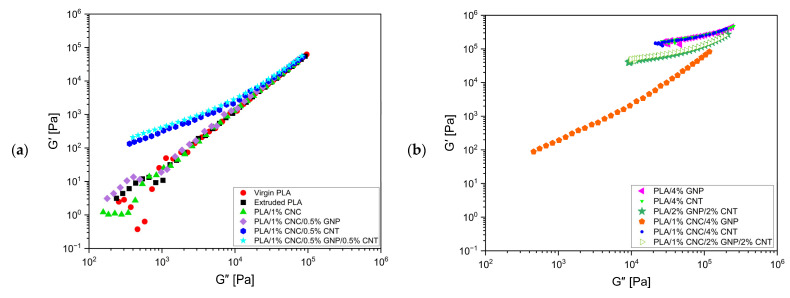
Cole–Cole plot of PLA-based composites at (**a**) low filler concentration and (**b**) high filler concentration.

At low filler loadings, the composites exhibited viscous-dominated behaviour and strong frequency dependence, indicating incomplete network formation (Figure 8a). With increasing concentration, moduli rose sharply, and several high-loading formulations, such as PLA/4% GNP, PLA/4% CNT, PLA/1% CNC/4% CNT, PLA/2% GNP/2% CNT, and PLA/1% CNC/2% GNP/2% CNT, displayed G′ > G″ without crossover (Figure 8b), signifying a pseudo-solid behaviour associated with continuous percolated, elastic-dominant networks [60]. This transition is clearly depicted in Figure 9, where G′ exceeds G″ and moving closer to the diagonal in high concentrations, reflecting the material’s shift to a solid-like characteristic. This confirms the formation of a percolation network, where the filler concentration surpasses the percolation threshold, leading to a solid-like characteristic (elastic-dominant material). These changes are consistent with the percolation theory, with the percolation threshold identified around 4%, where the material transitions from liquid-like to solid-like behaviour as the filler network becomes continuous (Figure 8 and Figure 9).

As shown in Figure 8c,d, the composites showed increasing shear-thinning behaviour with filler concentration, which is desirable for extrusion and FDM [18]. However, at excessive filler concentrations like PLA/4% CNT, aggregation intensifies filler–filler contacts, replacing polymer–polymer cohesion and generating stress concentrations at interfacial junctions. These locally rigid domains lead to brittle failure and undesirable processing challenges in rheology-sensitive applications like extrusion and FDM, including poor extrudability, weak interlayer adhesion, and nozzle clogging [21,63]. Therefore, optimal rheological performance of PLA-based composite is likely to occur near the percolation threshold, where a balanced combination of elasticity, toughness, and flowability, potentially making them suitable for high-quality FDM printing.

Rheological and cohesive energy data analyses demonstrate that filler type and loading govern the balance among elasticity, flow, and toughness in PLA-based composites. Increasing GNP and CNT contents enhances stiffness but reduces cohesion, while CNC improves dispersion and moderate brittleness at high filler levels. Optimal performance formation provides sufficient elasticity for shape retention and adequate toughness, which could support smooth FDM processing.

### 3.6. Correlation Between Structure and Multifunctional Properties

The collective analysis of morphological, rheological, electrical, and thermal results reveals a strong interdependence among filler dispersion, interfacial architecture, and overall multifunctional performance. SEM and TEM observations confirmed that the incorporation of CNC improved the distribution of GNP and CNT within the PLA matrix, promoting the formation of more interconnected conductive and thermally active pathways. CNC acted as a molecular bridge, enhancing matrix–filler coupling through hydrogen bonding and facilitating the formation of fibrous CNT pathways and planar GNP bridges for efficient charge and phonon transport. This hierarchical architecture is directly reflected in the rheological response, where increased storage modulus, cohesive energy density, and pronounced shear-thinning signify the development of a percolated elastic network in the melt state. The rheological resilience and controlled viscosity ensure that network formation does not compromise melt flow, potentially maintaining processability during extrusion and FDM printing.

The same interconnected filler network established through CNC-assisted dispersion governs both charge transport and phonon conduction. The correlated enhancement of electrical and thermal conductivities with filler content and hybridisation ratio confirms that both mechanisms originate from the same percolated structure. This correlation underscores that the continuity of the percolated network and the efficiency of interfacial coupling, dictate the multifunctional response of the CNC-assisted PLA composites. Overall, the synergistic interplay among nanoscale morphology, viscoelastic network formation, and dual (electrical and thermal) percolation explains the concurrent enhancement of functional properties and confirms the pivotal role of CNC in bridging structure–property relationships in the PLA hybrid composites.

### 3.7. Comparison of Results with Literature and Prototyping

As noted in the introduction, the literature indicates that numerous studies on PLA-based composites have primarily focused on enhancing electrical conductivity and mechanical performance through the incorporation of carbon-based fillers, particularly carbon nanotubes (CNTs) and graphene nanoplatelets (GNPs) (Table 1). Most studies have shown that increasing the concentration of carbon fillers improves these properties, and CNTs are particularly effective in enhancing electrical conductivity due to their 1D structure, ranging from $1.08\times 10^{-8}$ S/m (PLA/1.5% CNT) to 4.54 S/m (PLA/12% CNT) [25]. GNPs appear more effective in improving thermal conductivity with PLA/GNP composites thermal conductivity between 0.323 W/mK (PLA/3% GNP) [25] and 0.676 W/mK (PLA/12% GNP) [27]. It is important to note considerable variability in reported results especially for graphene which can be attributed to differences in the type and grade of used, including factors such as flake size, aspect ratio, surface area, oxidation level, and defect density. These characteristics critically affect the formation of conductive networks and, consequently, the percolation threshold. Nevertheless, the percolation threshold is generally obtained at filler concentrations around 6% or above and this pose challenges such as agglomeration and reduced processability.

Our approach, which combines CNC incorporation with three consecutive extrusion cycles to ensure homogeneous mixing without significantly altering the matrix properties, enables us to achieve a percolation threshold at a lower filler concentration. For example, PLA/1% CNC/4% achieved higher electrical conductivity (38.30 S/m) than PLA/12% CNT (4.54 S/m) and a comparable thermal conductivity with PLA/6% CNT [25]. PLA/1% CNC/2% GNP/2% CNT showed higher electrical conductivity (1.27 S/m) than PLA/12% GNP/12% CNT ($9.50\times 10^{-1}$ S/m) and thermal conductivity than PLA/3% GNP/3% CNT (0.270 W/mK) [25]. However, our GNP-system showed lower thermal conductivity than other literature probably due to smaller flake size affecting the concentration for reaching the percolation threshold. Although not presented here a better conductivity was achieved with a different relative graphene with bigger size. This work demonstrates that lower filler concentrations in PLA-based composites incorporated with CNC, such as PLA/1% CNC/4% CNT and PLA/1% CNC/2% GNP/2% CNT, can achieve comparable or superior electrical and thermal properties while circumventing common challenges of filler agglomeration and poor processability typically observed at higher filler concentrations. Such efficiency is particularly advantageous for sustainable smart packaging and other sensing applications, where minimising material usage and environmental impact without compromising functionality is critical.

**Figure 10:**
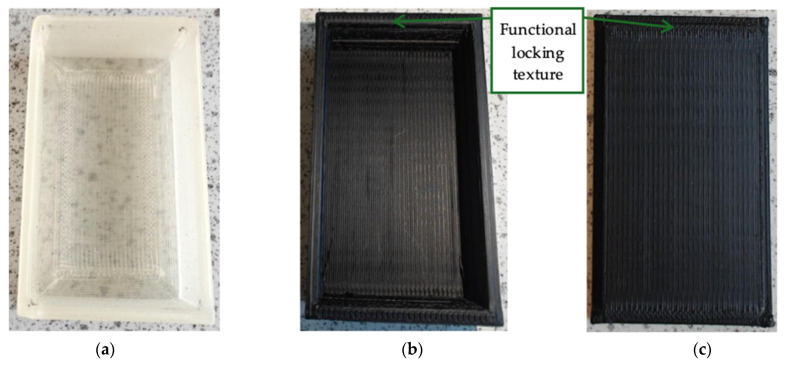
Typical prototypes: (**a**) box printed with PLA filament; (**b**) and (**c**) box and lid printed from the developed filament PLA/1% CNC/4% GNP, respectively.

To demonstrate the suitability of the developed composite filaments for FDM 3D printing, prototype boxes and lids were printed (Figure 10). The prototype box and lid printed from filament formulated with PLA/1% CNC/4% GNP (Figure 10b,c) have a functional locking texture. This edge texture was engineered to enhance fit, grip, and retention between the lid and the box, ensuring a secure and reliable closure rather than serving a purely decorative purpose.

## 4. Conclusions

This study successfully developed and characterised PLA-based composite filaments containing CNC, GNP, and CNT, highlighting the crucial role of CNC in enabling multifunctional performance and processability. SEM and TEM analyses revealed that CNC acts as a bio-based dispersion aid and network organiser, effectively mitigating GNP and CNT agglomeration and promoting a uniform, continuous hybrid network within the PLA matrix, thereby underpinning the observed enhancements in electrical and thermal performance. The highest conductivity (54.7 S/m) was achieved in PLA/1% CNC/4% CNT whereas PLA/1% CNC/2% GNP/2% CNT hybrid system exhibited the best thermal transport capability, reaching 0.279 W/mK at −20 °C, due to synergistic CNT- GNP integrations forming 3D heat-transfer pathways. However, the rheological analysis showed filler-dependent behaviour, where higher nanofiller loadings increased moduli and viscosity but lowered critical strain, indicating a stiffness–toughness trade-off suggesting. PLA/1% CNC/2% GNP/2% CNT emerged as promising formulation in this regard.

CNC synergistic interaction with GNP and CNT facilitated the development of biodegradable, high-performance, conductive PLA nanocomposites suitable for extrusion-based additive manufacturing, with potential application in smart packaging, sensors, and functional 3D-printed components. Future research should explore mechanical durability, print parameter optimisation, and large-scale filament manufacturing to fully exploit the industrial applicability of such bio-based hybrid systems.

## Figures and Tables

**Table 1:** Summary of related works on PLA nanocomposites: comparison of electrical conductivity, thermal conductivity, and critical strain data.

Materials	Electrical Conductivity (S/m)	Thermal Conductivity (W/mK)	Critical Strain (%)	Ref
PLA	$1\times 10^{-12}$	0.183	—	[25,27]
PLA/1.5% GNP	$1.5\times 10^{-12}$	—	—	[25]
PLA/3% GNP	$1.7\times 10^{-12}$	0.323	—	
PLA/6% GNP	$3.12\times 10^{-2}$	0.448	—	
PLA/9% GNP	$3.47\times 10^{-1}$	0.550	—	
PLA/12% GNP	6.27	0.664	—	
PLA/1.5% CNT	$1.08\times 10^{-8}$	—	—	
PLA/3% CNT	$1.40\times 10^{-2}$	0.231	—	
PLA/6% CNT	$6.57\times 10^{-1}$	0.232	—	
PLA/9% CNT	$9.40\times 10^{-1}$	0.268	—	
PLA/12% CNT	4.54	0.365	—	
PLA/3% GNP/3% CNT	$5.02\times 10^{-7}$	0.270	—	
PLA/6% GNP/6% CNT	$1.85\times 10^{-1}$	0.352	—	
PLA/12% GNP/12% CNT	$9.50\times 10^{-1}$	0.533	—	
PLA/12% GNPs	6.27	0.676	—	[27]
PLA/12% CNT	4.54	0.334	—	
PLA/6% GNP	$8.35\times 10^{-3}$	0.577	0.09	[23,28]
PLA/6% CNT	$2.10\times 10^{-2}$	0.303	—	
PLA/1.5% GNP/1.5% CNT	$5.40\times 10^{-7}$	0.3013	—	
PLA/1.5% GNP/4.5% CNT	$5.85\times 10^{-2}$	0.3779	0.32	
PLA/3% GNP/3% CNT	$3.60\times 10^{-2}$	0.4253	0.30	
PLA/4.5% GNP/1.5% CNT	$2.44\times 10^{-3}$	0.4692	0.25	
PLA/3% GNP	$9.00\times 10^{-8}$	—	—	[29]
PLA/7.5% GNP	0.20	—	—	
PLA/1% CNC/15% GNP	5.59	—	—	
PLA/4% CB	0.6	—	—	[30]
PLA/20% CB	14.3	—	—	
PLA/5.6% CNT	72.2	—	—	[31]
PLA/33.3 vol%(GNP/hBN)(50 GNP:50 hBN)	$8\times 10^{-4}$	2.77	—	[32]
PLA/4% GNP	$5.30\times 10^{-11}$	0.200	5.72	This work
PLA/4% CNT	30.70	0.226	1.04	
PLA/2% GNP/2% CNT	0.16	0.228	1.14	
PLA/1% CNC/4% GNP	$4.69\times 10^{-11}$	0.211	12.66	
PLA/1% CNC/4% CNT	38.30	0.245	0.51	
PLA/1% CNC/2% GNP/2% CNT	1.27	0.279	1.27	

“—” Data are not available. PLA: poly(lactic acid); CNC: cellulose nanocrystal; GNP: graphene nanoplatelet; CNT: carbon nanotube; CB: carbon black; hBN: hexagonal boron nitride. In this work, PLA nanocomposites were prepared by melt blending with three extrusion runs, whereas the literature samples were prepared by single-run melt blending, except Ref. [29], which employed solvent casting. Electrical conductivity and Thermal conductivity data from the literature were measured under ambient conditions. The reported data from this studied are those obtained at 25 °C, comparable to ambient temperature. Strain sweep tests were performed at 190 °C and 1 Hz to determine the critical strain at the end of the linear viscoelastic region, while rheological measurements in Ref. [28] were conducted at 220 °C and 1 Hz.

**Table 2:** Summary of the formulated PLA composites and labelling showing their compositions.

Composition Code (wt%)	PLA Content (wt%)	CNC Content (wt%)	GNP Content (wt%)	CNT Content (wt%)	Name
PLA	100	-	-	-	Reference
PLA/1% CNC	99	1	-	-	Mono-filler (CNC)
PLA/1% CNC/0.5% GNP	98.5	1	0.5	-	Bi-filler (CNC + GNP)
PLA/1% CNC/0.5% CNT	98.5	1	-	0.5	Bi-filler (CNC + CNT)
PLA/1% CNC/0.5% GNP/0.5% CNT	98	1	0.5	0.5	Tri-filler (CNC + GNP + CNT)
PLA/4% GNP	96	-	4	-	Mono-filler (GNP)
PLA/4% CNT	96	-	-	4	Mono-filler (CNT)
PLA/2% GNP/2% CNT	96	-	2	2	Bi-filler (GNP + CNT)
PLA/1% CNC/4% GNP	95	1	4	-	Bi-filler (CNC + GNP)
PLA/1% CNC/4% CNT	95	1	-	4	Bi-filler (CNC + CNT)
PLA/1% CNC/2% GNP/2% CNT	95	1	2	2	Tri-filler (CNC + GNP + CNT)

**Table 3:** Electrical conductivity of mono-, bi-, and tri-filler composites.

Composition Code (wt%)	Electrical Conductivity (S/m)
PLA	—
PLA/1% CNC	—
PLA/1% CNC/0.5% GNP	$8.52\times 10^{-11}\pm 1.00\times 10^{-11}$
PLA/1% CNC/0.5% CNT	$9.11\times 10^{-11}\pm 9.02\times 10^{-12}$
PLA/1% CNC/0.5% GNP/0.5% CNT	$6.20\times 10^{-11}\pm 9.87\times 10^{-12}$
PLA/4% GNP	$5.30\times 10^{-11}\pm 3.43\times 10^{-12}$
PLA/4% CNT	30.70 ± 2.1
PLA/2% GNP/2% CNT	0.16 ± 0.05
PLA/1% CNC/4% GNP	$4.69\times 10^{-11}\pm 4.85\times 10^{-12}$
PLA/1% CNC/4% CNT	38.30 ± 0.67
PLA/1% CNC/2% GNP/2% CNT	1.27 ± 0.24

“—” represents the data lower than the equipment detection limit.

**Table 4:** Thermal properties of virgin and extruded PLA after three consecutive extrusion cycles determined by TGA and DSC.

Materials	T_onset_ (°C)	T_max_ (°C)	T_g_ (°C)	T_m_ (°C)
Virgin PLA	341.10	361.22	58.72	154.08
Extruded PLA	341.34	359.77	57.48	150.94

Virgin PLA refers to the as-received poly(lactic acid) without prior processing, while extruded PLA denotes virgin PLA subjected to three consecutive extrusion runs under the same melt-blending conditions used for nanocomposite preparation. T_onset_ is the onset degradation temperature, T_max_ is the maximum degradation temperature, T_g_ is the glass transition temperature, T_cc_ is the cold crystallisation temperature, and T_m_ is the melting temperature.

**Table 5:** Yield stress, yield strain, critical strain, average storage modulus within the LVR, and cohesive energy density of neat PLA and its composites reinforced with CNC, GNP, and CNT. Data points are average of 3 replicates (*n* = 3).

Composition Code (wt%)	Yield Stress (Pa)	Yield Strain (-)	$\gamma_{c}$ (%)	${G_{c}}^{\prime }$ (Pa)	$E_{c}$ (J/m^3^)
Virgin PLA	2571.88	2.07	63.34	2427.87	487.05
Extruded PLA	2299.64	2.36	63.32	1923.87	385.68
PLA/1% CNC	1057.78	2.60	79.59	753.20	238.58
PLA/1% CNC/0.5% GNP	1709.54	2.07	63.32	1482.19	297.15
PLA/1% CNC/0.5% CNT	1680.80	1.86	7.98	2898.19	9.22
PLA/1% CNC/0.5% GNP/0.5% CNT	1707.11	1.65	7.95	4029.43	12.74
PLA/4% GNP	9085.03	1.69	5.72	22,089.56	35.77
PLA/4% CNT	16,924.93	0.76	1.04	226,452.63	12.18
PLA/2% GNP/2% CNT	10,384.81	1.19	1.14	78,757.90	5.20
PLA/1% CNC/4% GNP	2163.85	1.86	12.66	3311.70	26.54
PLA/1% CNC/4% CNT	15,334.67	0.54	0.51	235,215.63	3.04
PLA/1% CNC/2% GNP/2% CNT	9199.63	1.06	1.27	78,674.00	6.31

## Data Availability

The raw data supporting the conclusions of this article will be made available by the authors on request.

## Abbreviations

The following abbreviations are used in this manuscript:

CB	Carbon black
CNC	Cellulose nanocrystal
CNT	Carbon nanotube
EHP	Electrically heated parallel plate
FDM	Fused deposition modelling
GNP	Graphene nanoplatelet
hBN	Hexagonal boron nitride
LVR	Linear viscoelastic region
PLA	Poly(lactic acid)
SEM	Scanning electron microscopy
TEM	Transmission electron microscope
A	Cross-sectional area of filament
$C_{p}$	Specific heat capacity
$E_{c}$	Cohesive energy density
G’	Storage modulus
${G_{c}}^{\prime }$	Average storage modulus within the LVR
G”	Loss modulus
L	Length
R	Electrical resistance
α	Thermal diffusivity
γ	Absolute strain
$\gamma_{c}$	Critical strain
$\left|\eta^{*}\right|$	Complex viscosity
σ	Electrical conductivity
λ	Thermal conductivity
ρ	Bulk density
τ	Elastic stress
ω	Angular frequency
